# Assessment of Odour Emission During the Composting Process by Using Olfactory Methods and Gas Sensor Array Measurements

**DOI:** 10.3390/s25103153

**Published:** 2025-05-16

**Authors:** Mirosław Szyłak-Szydłowski, Wojciech Kos, Rafał Tarakowski, Miłosz Tkaczyk, Piotr Borowik

**Affiliations:** 1 Faculty of Building Services, Hydro and Environmental Engineering, Warsaw University of Technology, Nowowiejska 20 St., 00-653 Warsaw, Poland; wojciech.kos.dokt@pw.edu.pl; 2 Faculty of Physics, Warsaw University of Technology, ul. Koszykowa 75, 00-662 Warszawa, Poland; rafal.tarakowski@pw.edu.pl (R.T.); pborow@poczta.onet.pl (P.B.); 3 Forest Protection Department, Forest Research Institute, ul. Braci Leśnej 3, 05-090 Sękocin Stary, Poland; m.tkaczyk@ibles.waw.pl

**Keywords:** compost, electronic nose, odour emission, olfactometry, measurements, waste, VOC measurements

## Abstract

The final stage of green waste treatment typically occurs in composting plants, where waste is biologically stabilised through the activity of microorganisms. The composting process is accompanied by the emission of volatile organic compounds responsible for odour perception. Such nuisance odours are commonly regarded as atmospheric air pollutants and are subject to monitoring and legal regulation. Olfactometry remains the standard method for quantifying odours. Unfortunately, due to its dependence on human evaluators, it is often regarded as both labour-intensive and costly. Electronic noses are an emerging measurement method that could be used for such applications. This manuscript reports experimental measurements that were carried out at a composting facility specialising in the processing of biodegradable materials. VOC concentration was measured by the TSI OmniTrak™ Solution. The efficiency of the deodourisation process was evaluated by means of field olfactometry. A gas sensor array of a PEN3 electronic nose was used for the on-site measurements of emitted gas characteristics. A strong correlation between measurements by the three distinct techniques was confirmed. Three different phases of the composting process could be distinguished in the collected results.

## 1. Introduction

One of the fundamental objectives of waste management policy is the prevention of waste generation by addressing the problem at its source, recovering raw materials, reusing waste, and ensuring that the final disposal of residual waste is environmentally safe. Key priorities include the maximisation of resource recovery and minimisation of environmental harm, including the preparation of waste for environmentally sound landfilling. In Poland, this final stage of green waste treatment typically occurs in composting plants, where waste is biologically stabilised by the activity of microorganisms under aerobic or anaerobic conditions. This process is accompanied by the emission of odourants, the volatile organic compounds responsible for odour perception, which can spread over considerable distances. Odours are currently considered air pollutants and, in many countries, are subject to surveillance and legal regulation [1,2]. However, such regulations often focus on the degree of odour nuisance assessed by expert panels, which is insufficient. This approach captures only the perceived intensity of the nuisance experienced by individuals residing in the immediate vicinity of the odour source and fails to provide information on the specific types of emitted compounds, their concentrations, or other physicochemical characteristics of the odour.

Olfactometry remains the standard method for quantifying odours worldwide. However, due to its dependence on human evaluators, it is often regarded as labour-intensive and costly, particularly when applied on a regular basis [3]. Scaglia et al. [2] and Sironi et al. [4] argue that environmental impact studies of waste facilities should incorporate both olfactometric and chromatographic analyses, as well as electrochemical sensor method [5]. Different stages of the treatment process are associated with changes in gas composition, which in turn affect the quality and quantity of emitted odourants. Furthermore, the emission of specific groups of odorous compounds is determined by biochemical transformations occurring within the four phases of aerobic waste biodegradation: mesophilic, thermophilic, cooling and restructuring, and maturation [6,7]. The amount and composition of odour-active substances cause changes as the input waste stream progresses toward the parameters characteristic of the subsequent phase. It therefore appears particularly important to characterise the variability of odour concentration as a function of the day of the composting cycle. Operators of composting facilities require rapid, on-site methods for detecting process disturbances such as the onset of anaerobic conditions. Implementing real-time, continuous monitoring could also support operational decisions, for instance, by optimising the timing of compost pile turning [8]. An interesting research question is whether in situ olfactometric measurements can serve as a suitable method for monitoring and controlling the process, and for assessing its progress. It also remains an open issue whether such measurements can complement commonly used off-line optimisation methods that require waste sampling and time-consuming laboratory analyses.

Electronic noses (e-noses) may serve as a valuable complement to both olfactometric odour measurements and chromatographic analyses of volatile organic compounds (VOCs) [9,10]. They have demonstrated the ability to distinguish qualitative variations in the gases emitted, even when the VOC concentrations had very low differences which were associated with the type of compost and the particle size of the compost material [11]. Based on sensor arrays and pattern recognition algorithms, these devices enable the rapid, reproducible, and automated assessment of odour profiles in real time, without the need for sample collection or time-consuming laboratory procedures. Unlike dynamic olfactometry, e-noses do not require the involvement of human panellists, thereby eliminating subjectivity and limitations related to expert availability. Moreover, electronic noses can detect subtle changes in the composition of gas mixtures, which makes them useful tools for the real-time monitoring of biological processes such as composting or aerobic waste stabilisation. Their application can contribute to the early detection of process deviations, more effective odour control, and better optimisation of technological conditions.

Odour characterisation, both quantitative and qualitative, can be performed using sensorial methods, analytical techniques, or a combination of both. Sensorial approaches benefit from the superior sensitivity of the human nose compared to instrumental systems, yet their main limitation lies in the need for trained panellists to achieve consistent and reproducible outcomes. Conversely, analytical techniques eliminate human bias but tend to be less sensitive—especially when dealing with complex mixtures of odourants at low concentrations—and are generally unable to capture interactions between individual compounds [12].

Despite advances in instrumentation, the field monitoring of environmental odours remains a complex task. One major challenge lies in building reliable regression or classification models, which require not only a substantial dataset of sensor responses but also reference information regarding the odour intensity or source characteristics. While electronic noses operate autonomously to produce sensor data, gathering the associated reference variables still demands the prolonged presence of a human operator, which significantly limits the practicality of field applications—both in the initial model development and during subsequent validation and testing stages. Furthermore, end users often seek a straightforward decision-making tool, such as a function that can clearly indicate whether a given observation belongs to a specific odour category or quantify its nuisance potential. As noted by Nicolas [13], these limitations present significant barriers to the widespread deployment of electronic noses in environmental odour surveillance.

The concept of an electronic nose device [14] consists of the application of a series of non-specific gas sensors with an overlapping gas detection range. An electronic nose is a rapid, non-invasive, and intelligent online instrument. It comprises an array of carefully chosen sensors and a hydraulic system used for measured gas supply and sensor cleaning.

Various sensing methods based on electrical, thermal, optical, gravimetric, and electrochemical techniques have been developed [15,16,17,18,19]. Numerous commercial electronic nose devices are currently available on the market, applying various methods of gas measurements and various types of sensors. Probably the most common type of gas sensors used in electronic nose devices are metal oxide gas sensors [20], which are applied both in commercial [21] as well as custom-made devices [22].

In intensive food waste composting, a clear correlation has been observed between biological activity, quantified through the dynamic respiration index, and the generation of odourous compounds as detected by e-noses [23,24,25,26]. Although Gutiérrez et al. [27] investigated how total VOC concentrations change with compost maturity, they noted that e-noses lack the precision required to reliably estimate odour intensity and compost maturity.

The aim of this study was to explore and compare the effectiveness of three complementary approaches for characterising emissions from composting piles: field olfactometry, electronic nose, and VOC concentration measurements. Each of these methods offers distinct advantages in terms of response time, specificity, and potential for on-site deployment. By conducting a series of synchronised measurements throughout the composting cycle, we sought to assess the extent to which these techniques can capture the temporal dynamics of odour emissions and the underlying biological activity.

A key objective of the study was to determine whether the combined analysis of e-nose sensor responses and VOC concentrations could support the empirical definition and characterisation of biologically relevant composting phases. Furthermore, we examined the relationship between odour concentration (Cod) and the evolving chemical signature of emissions in order to evaluate whether odour-based indicators could serve as reliable proxies for process monitoring. The results contribute to the discussion on how real-time or near-real-time measurement techniques can supplement traditional, laboratory-based optimisation strategies, ultimately supporting more responsive and sustainable composting operations.

The contribution of this manuscript lies in its demonstration of a robust testbed for evaluating the interoperability and consistency of olfactometric, VOC, and e-nose measurements in a full-scale operational composting facility. Such field-level validation is critical for translating controlled-lab advancements into deployable environmental monitoring tools.

In our opinion, a complementary application of field olfactometry, electronic nose sensing, and PID-based VOC measurements can provide consistent and temporally resolved information on odour emissions during composting, sufficient to empirically distinguish biologically relevant process phases. We further assume that despite their methodological differences, these approaches will exhibit significant correlations, enabling their use as practical, field-deployable tools for routine environmental monitoring and process optimisation.

## 2. Materials and Methods

### 2.1. Composting Site

**Figure 1:**
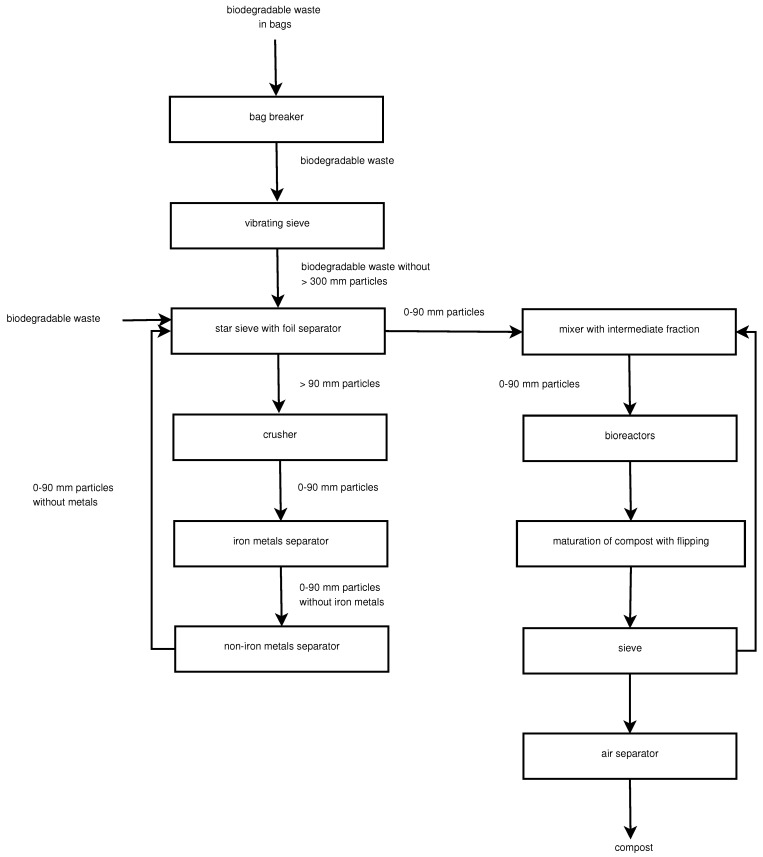
Block scheme for the management of biodegradable waste; 0–90 mm—sieve mesh size.

This research was carried out at a composting facility specialising in the processing of biodegradable materials, including dry leaves, grass clippings, and shredded woody biomass such as tree limbs and plant branches. The waste is treated through a series of sequential operations, utilising equipment such as cutters, vibrating and star sieves, crushers, and metal separators. The overall process also involves stages of material mixing and transport, as well as the application of an air separator for sorting and separating the input stream (Figure 1).

The waste material reflected the seasonal maintenance activities typically performed in early autumn and during the preparation of vegetation for winter. It consisted of a variety of plant residues generated through the upkeep of gardens, parks, and urban green areas. A substantial portion of the green waste comprises fallen leaves from trees and shrubs. In addition, small woody fragments produced during pruning operations were present, as such practices are common when preparing plants for colder months. Since lawn mowing was still ongoing, grass clippings also constituted a significant part of the composted material [28].

The composting process took place on an open composting pad and was carried out over several operational stages. The compost piles had a trapezoidal profile, with dimensions of approximately 4.5 m in width, 35 m in length, and 2 m in height. Each pile contained a waste mass ranging between 300 and 350 tonnes. Wheel loaders were used to periodically turn and aerate the piles, promoting adequate oxygen distribution and maintaining uniform airflow within the pile structure. The material was subjected to a maturation phase lasting from 10 to 14 weeks [29].

### 2.2. Measurements Methodology

The study was conducted on a total of 67 compost piles. The number of them analysed in each measurement campaign varied due to operational constraints at the composting site. Not all of the slots for piles in the composting site are occupied simultaneously, as free space is needed for turning and relocating the material. Therefore, only the piles present on-site during each series were included in the study: 25 piles on 03/15, 22 on 03/28, and 20 on 04/05. Air samples were collected from five designated points on the top surface (four corners and the geometric center of the pile plateau). Odour emission sampling was performed directly from the pile surface using a static chamber. Following the placement of the static chamber, a stabilisation period of 10 min was allowed to equalise internal pressure before sample collection. In total, 335 air samples were analysed throughout the experiment.

### 2.3. Gas Sampling

The static chamber employed in this study was constructed from an odour-inert material and featured a cylindrical body topped with a truncated hemispherical dome, having a radius of 20 cm. The overall height of the chamber was 30 cm, and it included a dedicated nozzle for connecting a sampling tube. The effective internal volume—defined as the air space above the emission surface—was calculated to be 0.04 m^3^.

A significant challenge in the determination of odour concentration is the potential interference from ambient background odours present during sampling. To mitigate this issue, the experimental setup utilised stainless steel components, selected for their resistance to odour retention and ease of sterilisation. Air sampling and transfer were performed exclusively through virgin polytetrafluoroethylene (PTFE) tubing, ensuring an uncontaminated sampling pathway. Furthermore, a carbon filter was used to purify the technical air supply, thereby eliminating residual odours and preventing background contamination.

In this context, “background odour” refers to volatile compounds naturally present at the sampling site. The methodology applied here involved capturing a fraction of the emitted air and quantifying its odour concentration at the outlet. Although the sampling system was designed to exclude background odours, it must be acknowledged that the detection threshold of the analytical method may fall below the ambient odour levels inherently present in the environment [28].

### 2.4. Measurements of Weather Conditions

Temperature (T, °C) and relative humidity (RH, %) were measured using a Rotronic HydroPalm psychrometer with a HygroClip2 HC2-S3 sensor (Rotronic AG, Bassersdorf, Switzerland). Measurements were conducted at a height of 1.5 m. Atmospheric pressure, dew point, and wind speed (m/s) were measured using a Kestrel 5500 Weather Meter (Kestrel Instruments, Boothwyn, PA, USA).

### 2.5. Measurements of Concentration of Volatile Organic Components

VOCs were measured by the TSI OmniTrak™ Solution (Shoreview, MN, USA) with VOCs (ppb) module. The device is equipped with a 10.6 eV Photo Ionisation Detector (PID) sensor, offering a measurement range of 0–20,000 ppb and a resolution of 1 ppb. The response time of the sensor is 15 s. Measurement specifications are valid under ambient conditions of temperature 21 ± 5 °C, pressure 98.6 ± 5 kPa, and relative humidity 50 ± 10%.

### 2.6. Measurements of Odour Concentration

The efficiency of the deodourisation process was evaluated by means of field olfactometry. The measurements were conducted using a Scentroid SM100 olfactometer (IDES Canada Inc., Toronto, ON, Canada) in combination with a static sampling chamber. The portable olfactometer allows for the dilution of odourous air with clean, filtered air and the quantification of odour concentration on-site. Results were expressed in odour units per cubic metre (ou/m^3^) in accordance with the European standard PN-EN 13725:2022 [30].

The Scentroid SM100 device enables measurements within a broad range from 2 to 30,000 ou/m^3^ [31]. In comparison, the widely used Nasal Ranger provides a narrower measurement range and lower precision [32]. Prior to sampling, panellists’ olfactory sensitivity was verified using the Sniffin’ Sticks Test (SST), following ISO 13301:2018 recommendations [33].

At each sampling site, four individual olfactometric measurements were performed. According to the PN-EN 13725:2022 standard, the final odour concentration was calculated as the geometric mean of these individual values [29].

### 2.7. Measurements by Electronic Nose Gas Sensor Array

A commercially available PEN3 electronic nose device (Airsense Analytics GmbH, Schwerin, Germany) [21] was used in the reported experiment. The electronic nose consists of a hydraulic gas extraction and sampling system, a sensor array consisting of 10 metal oxide semiconductor sensors forming a detection unit, and proprietary software for data acquisition. The electronic nose sensors operate at high temperatures (150 to 500 °C) and respond to the presence of various gaseous chemical components. In Table 1 is a list of PEN3 sensors and the characteristic gases which they can detect.

During the measurement procedure, we followed the manufacturer’s recommendation, according to which, before performing a series of measurements, the electronic nose was warmed up for 10 min. Before each measurement cycle, air cleaned with an activated carbon filter was blown through the device to clean the gas transfer tubes and sensors. During the measurement phase, gas was aspirated into the sensor chamber at a constant flow rate. The measurement cycle lasted 2 min, and the sensor response signals were recorded every second.

The sensors’ response to the presence of the measured gas is represented as the sensor conductivity normalised to the baseline value (G/G_0_). The baseline level G_0_ is the sensor conductance determined at the beginning of each measurement cycle when the sensors were exposed to clean air conditions. The characteristic recommended by the manufacturer of the PEN3 e-nose is the final steady state value of the response curve G_*ss*_/G_0_.

Many authors have proposed various features that can be extracted from the dynamic characteristics of the sensor response [34] and consequently considered as characteristics of the sensor response to a given gas composition. According to the manufacturer’s recommendation, the most basic feature that can be used is the steady state level of the sensor’s response that is reached after the sensor’s exposure to the measured gas. The transient sensor response lasted up to about 1 min, and since in our measurements we collected data for up to 2 min, for further analysis, we used the average of the sensor response during the second minute of the measurements. This allowed us to reduce the noise level of the response.

## 3. Results and Discussion

### 3.1. Measurement of Weather Conditions

- On 15 March 2025: The temperature rose from approximately 0 °C at 8:00 to 6 °C at 12:00. Humidity decreased from 82% to 45%. The dew point ranged from −2 °C to 2 °C. Atmospheric pressure increased from 1005 hPa to 1009 hPa. The average wind speed ranged from 3.0 m/s to 4.3 m/s.
- On 28 March 2025: The temperature rose from approximately 8 °C at 8:00 to 14 °C at 12:00. Humidity decreased from 65% to 45%. The dew point ranged from 3 °C to 6 °C. Atmospheric pressure increased from 1012 hPa to 1016 hPa. The average wind speed ranged from 3.6 m/s to 4.2 m/s.
- On 5 April 2025: The temperature rose from approximately 3 °C at 8:00 to 8 °C at 12:00. Humidity decreased from 85% to 60%. The dew point ranged from 1 °C to 4 °C. Atmospheric pressure increased from 1008 hPa to 1012 hPa. The average wind speed ranged from 1.2 m/s to 2.2 m/s.

### 3.2. Measurements of VOC and Odour Concentration

**Figure 2:**
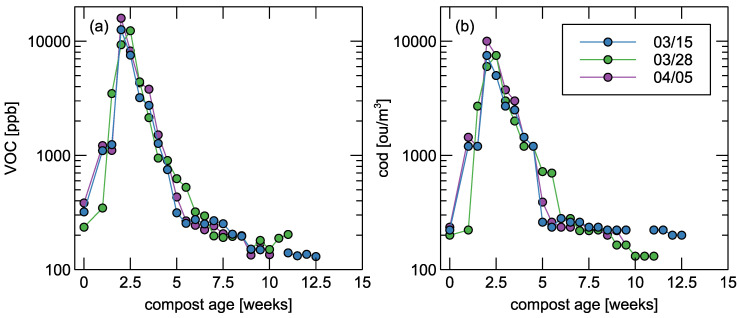
Concentration of VOC (**a**), and Cod (**b**) versus measured compost pile age. Volatile organic carbon compounds concentration was measured by PID sensor, while odour concentration—field olfactometry. Date of measurement series is indicated in the legend.

Figure 2 contains the results of VOC and odour concentration during each week of composting, within three measurement series.

To assess whether VOC levels differed significantly across the three composting trials, one-way ANOVA and a Kruskal–Wallis test were performed [35]:

- ANOVA: F(2,62)=0.135, p=0.874;
- Kruskal–Wallis: $\chi^{2}\left(2\right)=0.434$, p=0.805.

Both tests did not reveal statistically significant differences, indicating that there is no sufficient evidence to reject the null hypothesis. This suggests that the temporal VOC emission patterns are consistent across series.

Pearson’s correlation coefficients (r) were calculated to assess the linear relationship between the concentrations of volatile organic compounds and odour concentrations [35]. Correlations were computed separately for each measurement series, and statistical significance was evaluated using corresponding *p*-values. Strong Pearson correlations were observed between VOC levels and odour concentration (Cod) within each series: 03/15: r=0.988; 03/28: r=0.993; 04/05: r=0.981. This highlights the potential of using VOC measurements as reliable indicators of odour emissions during composting. The extremely low *p*-values (*p* less than $10^{-9}$) confirm that the observed correlations are highly unlikely to have occurred by chance, particularly in the 04/05 series where the confidence interval is remarkably tight.

### 3.3. Distinction of Three Phases of the Composting Process

According to the results, the composting process can be divided into three biologically motivated phases: Phase 1 (initial decomposition, Weeks 0–2), Phase 2 (active degradation, Weeks 2–5), and Phase 3 (maturation and stabilisation, Weeks 5+). Within each phase, the average weekly change in concentrations of volatile organic compounds (VOCs) and odour units (Cod) was calculated for each of the three independent series (03/15, 03/28, and 04/05).

After segmenting the composting process into distinct temporal phases, the average weekly change (emission gradient) in VOC and Cod concentrations was calculated within each phase for all three-measurement series. The trend analysis illustrates both the direction and the magnitude of odour emission changes over time. Table 2 presents the emission gradients (expressed in ppb/week for VOC and ou/m^3^/week for Cod), reflecting the rate and direction of change in odour-related parameters.

As seen in Table 2, both VOC and odour concentration increased rapidly in Phase 1, decreased markedly in Phase 2, and stabilised in Phase 3 across all trials.

- Phase 1, a sharp increase in both VOC and Cod concentrations was observed across all series, with VOC levels rising by +3025 to +5172 ppb/week, and Cod increasing by +1933 to +3255 ou/m^3^/week. This period corresponds to the intense microbial breakdown of readily degradable organic matter and the onset of thermophilic conditions.
- During Phase 2, all series exhibited a substantial decline in emissions, with VOC gradients ranging from −1448 to −2578 ppb/week and Cod from −880 to −1602 ou/m^3^/week. This decline indicates the progression toward stabilisation and the depletion of easily degradable substrates.
- Finally, Phase 3 showed minimal changes in emissions, with average gradients close to zero for both VOC (−15 to −35 ppb/week) and Cod (−5 to −49 ou/m^3^/week). This plateau suggests the compost had reached a state of biological maturity and odourant release had largely subsided.

**Figure 3:**
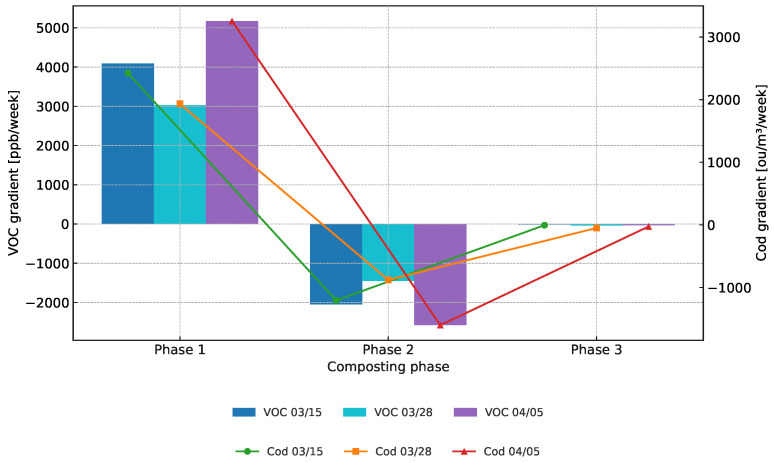
Comparison of average weekly changes in VOC and Cod across composting phases.

Figure 3 illustrates the temporal dynamics of VOCs and Cod, displaying the trends observed during composting phases.

These results confirm the expected emission dynamics in composting systems: there is an initial emission surge, followed by a marked decrease, and finally stabilisation. The consistency of this pattern across all series supports the robustness of the temporal phase model in describing odour emissions in composting environments.

During the examinations of Kim et al. [36], the total concentration of volatile organic compounds in composting piles gradually decreased during the process from 45 to 35 mg/kg after three weeks of composting. This observed reduction resulted from the volatilisation and biodegradation of VOCs under composting conditions [36]. However, in the study by Sánchez-Monedero et al. [37], in the case of mature compost, after eight weeks of treatment, an increase in the total VOC content to 38 mg/kg was observed. This phenomenon was associated with the enrichment of organic matter in the mature compost, resulting from the screening performed prior to open-air storage. The emission of volatile organic compounds (VOCs) was primarily influenced by microbial activity and the transient development of anaerobic microenvironments within the compost pile. The maximum VOC emission was observed at the end of the second week of the composting process. Several studies have consistently demonstrated a significant reduction in VOC emissions throughout the stages of municipal solid waste (MSW) processing. According to Scaglia et al. [2], Delgado-Rodríguez et al. [38], and Gallego et al. [39], VOC emissions can decrease by as much as 95% from the initial reception and sorting of MSW to the final storage of stabilised compost. Smet et al. [40] conducted pilot-scale research comparing a combined anaerobic/aerobic composting process (CCP) and a conventional aerobic composting process (ACP). They reported total VOC emissions of 590 g/Mg of waste in the case of ACP, while for the CCP, emissions were measured at 217 g/Mg during the initial anaerobic phase and only 0.3 g/Mg during the subsequent aerobic phase. In a related study, Cadena et al. [41] investigated VOC emissions during a two-week decomposition phase conducted in enclosed composting reactors equipped with controlled aeration, moisture regulation, and off-gas collection systems, followed by gas treatment via a wet scrubber and a biofilter. The subsequent curing phase lasted 6–8 weeks and was carried out in forced-aerated piles exposed to the atmosphere, without gas collection. The recorded VOC emissions in this system were approximately 200 g/Mg. Kumar et al. [42] reported that alcohols were the predominant class of volatile organic compounds (VOCs) emitted from compost piles, regardless of their maturation stage. The highest overall emissions were associated with younger composting piles (3–6 days old). Moreover, average emissions of non-alcohol VOCs were approximately twice as high in these younger piles compared to both freshly tipped piles and more mature composting stages. These findings are consistent with the present study, in which the most intensive VOC emissions were observed during the initial stage of the composting process. According to Nordahl et al. [43], the average VOC emission during the composting of yard waste was $5.23\;\times \;10^{-4}\;\mathrm{kg}\;\mathrm{VOC}\;{\mathrm{kg}}^{-1}$ of wet feedstock, whereas for the composting of the organic fraction of municipal solid waste, it reached $1.71\;\times \;10^{-3}\;\mathrm{kg}\;\mathrm{VOC}\;{\mathrm{kg}}^{-1}$ of wet feedstock. These results were obtained under laboratory-scale composting conditions. In the present study, VOC emissions ranged from $1.57\;\times \;10^{-8}$ to $1.86\;\times \;10^{-8}\;\mathrm{kg}\;\mathrm{VOC}/\mathrm{kg}\;\mathrm{wet}\;\mathrm{feedstock}$.

Biasioli et al. [44] reported that freshly stacked compost, composed entirely of the organic fraction, exhibited an odour concentration of approximately 750 ou/m^3^. After 20 days of aeration, once the Gore-Tex membrane was removed, the odour concentration increased to 1300 ou/m^3^. Similarly, in a recent study by Szyłak-Szydłowski and Kos [29], the odour concentration of fresh compost was measured at 750 ou/m^3^ and rose to 1250 ou/m^3^ after two weeks of composting. In the present study, the initial odour concentration ranged from 200 to 235 ou/m^3^. The maximum value of odour concentration—−10,000 ou/m^3^—was achieved during the second week of composting. Fischer et al. [45] reported that diffuse gas emissions that occured during the turning of open compost piles were associated with odour concentrations exceeding 3000 ou/m^3^. During the first week, odour levels peaked at approximately 8000 ou/m^3^, but declined to around 4000 ou/m^3^ by the second week.

A comparable emission pattern was documented by Rincón et al. [46], who investigated the composting of anaerobically digested sewage sludge. They found that approximately 40% of the total mass of volatile emissions occurred during the initial eight days of the active composting stage, referred to as the “early active phase” [28]. Scaglia et al. [2] evaluated the odour impact of waste at three distinct composting stages: immediately after deposition (time zero), after four weeks, and after thirteen weeks. The corresponding odour concentrations recorded were 28,546 ou/m^3^, 4902 ou/m^3^, and 2569 ou/m^3^, respectively.

According to Defoer et al. [47], the highest odour concentrations in compost piles were observed during the first week of the biostabilisation process. In their study on composting of vegetable, fruit, and garden (VFG) waste, a strong linear relationship was observed between total VOC concentration and odour intensity—the odour concentration also showed a strong correlation with the presence of esters and ketones. In the present study, a strong correlation was also observed between VOC concentrations and the olfactory parameter of odour concentration. In contrast, Sekeran et al. [48] and Tin Lee [49] reported a reduction in odour intensity within the first three weeks of composting, whereas in the control samples, this decline occurred only after five weeks. Similarly, Khalil et al. [50] noted a decrease in odour nuisance after five weeks in compost inoculated with effective microorganisms compared to seven weeks in the untreated control.

### 3.4. Measurements by the Electronic Nose

**Figure 4:**
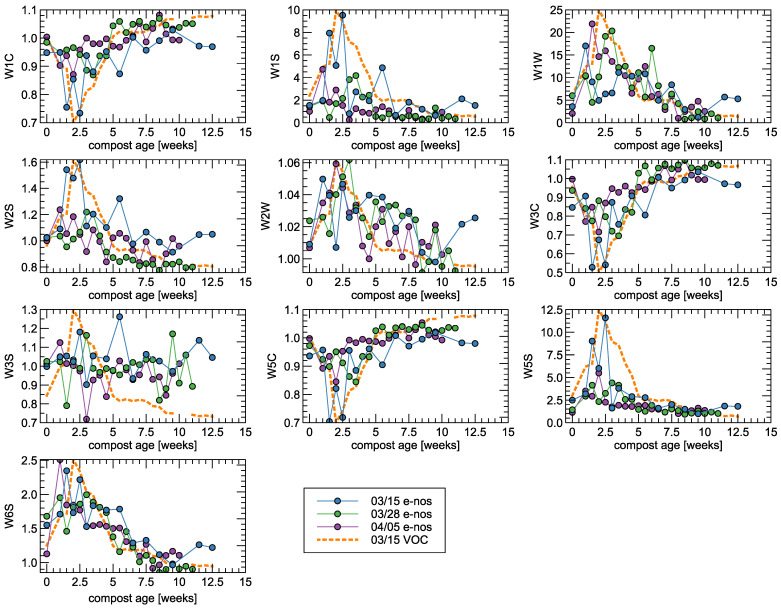
Stationary state level of the sensor response (G/G_0_) after exposure to the measured gas versus maturity (age) of the measured compost pile. The sensor type is indicated in the y-axis of the subfigures. Three series of measurements as indicated in the legend. A dashed line is presented for the eye guide and represents the VOC concentration on a logarithmic scale. For the sensors, whose response is in a negative direction, the VOC curve is presented with a changed sign to more easily follow the common trends in the data.

In Figure 4 we present the response of the electronic nose sensors exposed to compost piles of various ages (maturity). In the same figure, we present a curve representing measurements of VOC, presented already in Figure 2, with the purpose of highlighting similar trends in both types of measurements. Since the VOC measurements in all measurement series were very close, we presented a series for only one date of measurement. The used PEN3 electronic nose device is based on metal oxide sensors, for which the usual response characteristics are not linear with gas concentration and often exhibit rather exponential reactions. For that reason, we chose to present the VOC concentration curve in a logarithmic scale, which is more appropriate for such comparisons.

Based on the signal intensity of ten sensors incorporated in the electronic nose, distinct changes in the composition of volatile gases were observed throughout the composting period.

During Phase 1 (Weeks 0–2), most sensors exhibited high signal intensities, indicating strong emissions of volatile compounds typically associated with anaerobic microbial activity. The W1S sensor, which is sensitive to methane and a broad range of organic gases, showed a particularly sharp peak. Similarly, W1W responded strongly to the presence of inorganic sulphur compounds, such as hydrogen sulphide and sulphur-containing VOCs, which are byproducts of early protein degradation. W5S also showed a high and broad response, indicating the presence of reactive gases such as nitrogen oxides. W6S detected hydrogen emissions, which are expected in early anaerobic phases. Strong responses from W3S and W5C further support the presence of methane, alkanes, and various aromatic hydrocarbons. These sensor responses suggest that Phase 1 is characterised by low oxygen availability and intense anaerobic decomposition processes.

This interpretation is strongly supported by the direct measurement of VOC concentrations, which reached their highest levels during this initial phase. A sharp VOC peak was recorded in early weeks, and the elevated VOC values are consistent with the pronounced sensor signals, confirming that Phase 1 is the most odourous and chemically active stage of the composting cycle.

In Phase 2 (Weeks 2–5), the signals from W1S, W1W, and W5S decreased noticeably, indicating a reduction in anaerobic emissions such as H_2_S and methane. At the same time, W2S, W2W, W1C, and W3C showed increased and more stable signals. These sensors are sensitive to alcohols, phenols, ammonia, and other oxygenated compounds. This phase likely reflects the dominance of aerobic microbial activity, with increased oxygen availability promoting the breakdown of organic matter into less odourous and more oxidised compounds. W5C activity in this phase may indicate the continued presence of alkanes and less polar VOCs.

VOC measurements during Phase 2 exhibited a moderate but still significant presence of volatile compounds. While concentrations were lower than in the initial phase, their persistence confirms ongoing biological activity and the release of degradation products such as alcohols, aldehydes, and organic acids. The pattern of VOC reduction aligns closely with the e-nose signal stabilisation, suggesting a progressive shift from anaerobic to aerobic decomposition pathways.

By Phase 3 (Week 5 onward), most sensor responses declined or reached a steady plateau. Sensors previously active during anaerobic phases (W1S, W1W, W5S, W6S) showed minimal activity, reflecting the completion of the most intensive decomposition processes. However, a moderate response remained from sensors W1C, W2W, and W3C, indicating trace levels of aromatic compounds and ammonia, which may be associated with the final stages of lignin transformation or residual microbial activity. Overall, this phase is marked by biological stabilisation and minimal emissions of odourous gases, confirming the transition toward mature compost.

VOC concentrations during this final stage were consistently low and showed minimal fluctuations. The decline in volatile emissions confirms the advanced degree of compost maturation and aligns with low e-nose activity. Together, these indicators reinforce that Phase 3 is the least chemically reactive and least odourous phase, suitable for final use or composting.

### 3.5. Correlation Between the Measurements Methods

**Figure 5:**
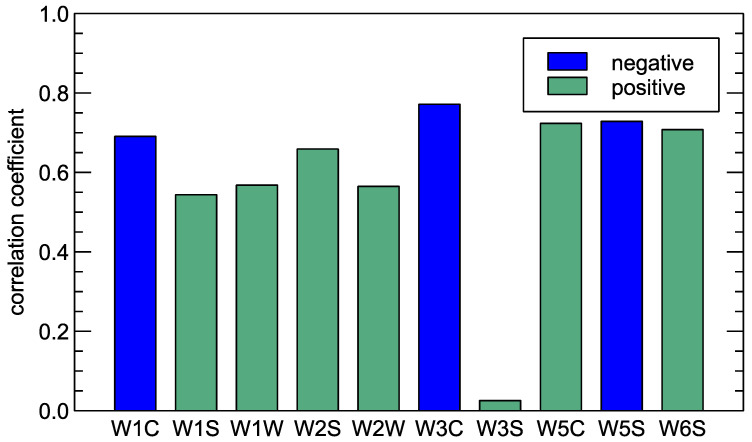
Correlation coefficient between sensor response and logarithm of VOC concentration. Absolute value of the correlation is presented, and its sign is indicated by the bar’s colours.

In Figure 4, one can observe the qualitative agreement of the results obtained by two methods of measurement, as the collected data follow similar patterns for some of the sensors. To some extent, we can quantify the interrelationship between the two types of results by calculating the correlation coefficient. In Figure 5 and Table 3, we present a comparison of the Pearson correlation coefficients for the responses of all sensors of PEN3 electronic nose and VOC concentration measured by the olfactometry method. The second quantity was taken on a logarithmic scale as explained above. The average over three collected series of measurements is presented.

As one can notice, we cannot observe a correlation between the W3S sensor response and the concentration of VOC. It can be observed in Figure 4 that this sensor does not exhibit any significant response to the measured conditions. The collected response signal is close to one (the response does not differ from clean air conditions), with some variability in the data.

The strongest correlation coefficient of the order of −0.77 for the W3C sensor can be confirmed with the main gas target presented in Table 1, which is “Ammonia, often used as a sensor for aromatic compounds”. Also, the responses of sensors W5S, W5C, W6S, and W1C are correlated with VOC concentration, with a magnitude of correlation coefficient above 0.7.

D’Imporzano et al. [51] and Orzi et al. [52] investigated the relationship between VOC emissions, odour perception (assessed via electronic nose and olfactometry), and biological activity during organic waste treatment processes. In a pilot-scale study on food waste composting, D’Imporzano et al. [51] identified a strong correlation between odourous compounds detected by an electronic nose and the Dynamic Respiration Index (DRI), a key indicator of microbial activity. Similarly, Orzi et al. [52] examined the association between odour emissions (quantified both via olfactometry and electronic nose), VOC concentration (measured with GC-MS), and biological activity (assessed through aerobic and anaerobic indices) during various stages of the anaerobic digestion of municipal organic waste. Their findings indicated that as biological stability progressed—from raw to post-digested material—odour emissions decreased, as measured by olfactometry. However, they did not observe a clear correlation between total VOC concentrations and olfactometric odour levels [53].

It should be admitted that in the presented research, the electronic nose device application was limited to the collection of responses of gas sensors exposed to the odour emitted by composting piles. The automatisation of the process of gas supply, sensor cleaning, and data collection by the PEN3 device allowed the measurements to be performed in the composting plant. We have not performed a sophisticated analysis of gas sensor data applying machine learning models, as this is common practice. The usual problem in other electronic nose research is the differentiation between sample categories. Instead, the present study focuses on the regression problem in the electronic nose gas sensors’ responses, which we were able to correlate with the concentration of the VOCs measured by olfactometry. What is more, in our opinion, a more sophisticated comparison of the results should consider not only the concentration of the VOCs but also the possible change in the chemical composition of the emitted gases in various stages of the composting process. Unfortunately, at this stage of our research, we were not able to perform an analysis of chemical components by gas chromatography methods, which we hope will be possible in future experiments. Due to a limited amount of collected data, we limited our analysis to correlation, without building more sophisticated machine learning models. In our opinion, at this experimental stage, only rather quantitative results were obtained.

## 4. Summary and Conclusions

Odours emitted during the compost maturing process are regarded as atmospheric air pollution and are subject to monitoring and legal regulations. This requires the odour nuisance to be measured, which is usually performed by expert panels. Olfactometry remains the standard method for quantifying odours.

This manuscript reports measurements performed at a composting facility specialising in the processing of biodegradable materials. The composting process took place on an open composting pad. The material was subject to maturation lasting from 10 to 14 weeks. Odour emission sampling was performed directly from the pile surface using a static chamber. Air sampling and transfer were performed exclusively through virgin polytetrafluoroethylene tubing, ensuring an uncontaminated sampling pathway. A carbon filter was used to purify the technical air supply.

Volatile organic compounds were measured using TSI OmniTrak™ Solution. A Scentroid SM100 field olfactometer was used for the assesement of odour concentration. A PEN3 electronic nose was used for complementary measurements with 10 metal oxide sensors.

The composting process showed highly repeatable emission patterns for both VOCs and odour intensity. All trials followed a consistent pattern of an increase, decrease, and stabilisation of emissions. The strong correlation between VOC and Cod suggests that VOC measurements can serve as a practical proxy for odour monitoring in composting systems.

It was also confirmed that the measurements via electronic nose are strongly correlated with the measurements of VOC—the correlation between sensor responses and the logarithm of VOC concentrations reached |r| > 0.7 for several sensors–confirming that the e-nose technique can be viewed as a complementary method of assessment and a way to monitor odour intensity.

Three phases of the composting process can be distinguished in the results obtained by all the measurement methods. The highest concentrations of VOCs and odour (Cod) were recorded at the end of the second week of the composting process, which coincides with intense microbiological activity and the transient occurrence of anaerobic zones. This could be also attributed to an intense microbial breakdown of organic matter. In the following 3 weeks, a substantial decline in emissions was observed, which was caused by the depletion of easily degradable substrates. After 5 weeks of composting, the minimal changes in emissions indicated that the compost had reached a state of biological maturity and odourant release had largely subsided.

This work demonstrates the readiness of the field to combine olfactometric, VOC, and e-nose methods under operational composting conditions. The findings provide a data-backed rationale for deploying low-cost, semi-automated solutions in odour surveillance frameworks and optimising composting protocols in real time.

## Figures and Tables

**Table 1:** The list of PEN3 electronic nose sensors.

Sensor	Main Gas Targets
W1C	Aromatic organic compounds.
W1S	Sensitive to methane (environmental). Can detect a broad range of organic compounds.
W1W	Detects inorganic sulphur compounds, e.g., H_2_S. Also sensitive to many terpenes and sulphur-containing organic compounds.
W2S	Detects alcohol, is partially sensitive to aromatic compounds, and has a broad range.
W2W	Aromatic compounds and sulphur organic compounds.
W3C	Ammonia, often used as a sensor for aromatic compounds.
W3S	Reacts to high concentrations of methane (very selective).
W5C	Alkanes, aromatic compounds, and less polar organic compounds.
W5S	Has a broad range of sensitivity, reacts to nitrogen oxides, and is very sensitive with negative signals.
W6S	Detects mainly hydrogen gas and is selective (breath gases).

**Table 2:** Average weekly change (gradient) in VOC and Cod concentrations across composting phases.

Phase	VOC (ppb/week)			Cod (ou/m^3^/week)		
	**03/15**	**03/28**	**04/05**	**03/15**	**03/28**	**04/05**
Phase 1	+4093	+3025	+5172	+2426	+1933	+3255
Phase 2	−2048	−1448	−2578	−1207	−880	−1602
Phase 3	−15	−35	−30	−5	−49	−26

**Table 3:** Pearson correlation coefficient between response of each sensor exposed to compost pile of various maturities, and logarithm of concentration of VOC.

W1C	W1S	W1W	W2S	W2W	W3C	W3S	W5C	W5S	W6S
−0.69	0.54	0.57	0.66	0.56	−0.77	0.026	0.72	−0.73	0.71

## Data Availability

The original contributions presented in the study are included in the article, and further enquiries can be directed to the corresponding author.
